# Supplementary material for: Experiences of transgender and non-binary youth accessing gender-affirming care: A systematic review and meta-ethnography
Source: PLoS One. 2021 Sep 10;16(9):e0257194. doi: 10.1371/journal.pone.0257194 (PMC8432766; doi:10.1371/journal.pone.0257194)
Supplement: S1 File — (DOCX) [file pone.0257194.s001.docx]

## S1 File. ENTREQ checklist.

### Tong A, Flemming K, McInnes E, Oliver S, Craig J. Enhancing transparency in reporting the synthesis of qualitative research: ENTREQ. BMC Med Res Methodol. 2012;12(1):181.

| No | Item | Guide and description | Response (Page No. in manuscript) |
| --- | --- | --- | --- |
| 1 | Aim | State the research question the synthesis addresses. | Aim:  Systematically search, retrieve, appraise and synthesise the qualitative empirical literature on the experiences of young transgender and non-binary youth with accessing healthcare.  (Introduction p.7) |
| 2 | Synthesis methodology | Identify the synthesis methodology or theoretical framework which underpins the synthesis, and describe the rationale for choice of methodology *(e.g. meta-ethnography, thematic synthesis, critical interpretive synthesis, grounded theory synthesis, realist synthesis, meta-aggregation, meta-study, framework synthesis).* | Meta-ethnography  (Abstract, Methods p2, p8) |
| 3 | Approach to searching | Indicate whether the search was pre-planned (*comprehensive search strategies to seek all available studies)* or iterative (*to seek all available concepts until they theoretical saturation is achieved)*. | Pre-planned and search strategy described in methods section, subheading search strategy (p2,8). |
| 4 | Inclusion criteria | Specify the inclusion/exclusion criteria *(e.g. in terms of population, language, year limits, type of publication, study type).* | See “Inclusion and exclusion criteria” (Method,p8/9). |
| 5 | Data sources | Describe the information sources used (e.g. *electronic databases (MEDLINE, EMBASE, CINAHL, psycINFO, Econlit), grey literature databases (digital thesis, policy reports), relevant organisational websites, experts, information specialists, generic web searches (Google Scholar) hand searching, reference lists)* and when the searches conducted; provide the rationale for using the data sources. | See “Search strategy (Method,p8). |
| 6 | Electronic Search strategy | Describe the literature search *(e.g. provide electronic search strategies with population terms, clinical or health topic terms, experiential or social phenomena related terms, filters for qualitative research, and search limits)*. | See “Search strategy” (Methods, pp.8; Supplementary file shows search strategy |
| 7 | Study screening methods | Describe the process of study screening and sifting *(e.g. title, abstract and full text review, number of independent reviewers who screened studies).* | See “Screening” (Method, p.9). |
| 8 | Study characteristics | Present the characteristics of the included studies *(e.g. year of publication, country, population, number of participants, data collection, methodology, analysis, research questions).* | See “Characteristics of included studies”. This information is included in table form p12-19 |
| 9 | Study selection results | Identify the number of studies screened and provide reasons for study exclusion *(e,g, for comprehensive searching, provide numbers of studies screened and reasons for exclusion indicated in a figure/flowchart; for iterative searching describe reasons for study exclusion and inclusion based on modifications t the research question and/or contribution to theory development).* | See “Results” (Results, p.11). |
| 10 | Rationale for appraisal | Describe the rationale and approach used to appraise the included studies or selected findings *(e.g. assessment of conduct (validity and robustness), assessment of reporting (transparency), assessment of content and utility of the findings).* | See “Results p20 and methods p8). |
| 11 | Appraisal items | State the tools, frameworks and criteria used to appraise the studies or selected findings *(e.g. Existing tools: CASP, QARI, COREQ, Mays and Pope* [[25](http://www.ncbi.nlm.nih.gov/pmc/articles/PMC3552766/#B25)]*; reviewer developed tools; describe the domains assessed: research team, study design, data analysis and interpretations, reporting).* | See “Quality appraisal” (Method, pp.9).  Critical Appraisal Skills Programme. CASP Qualitative Checklist: Critical Appraisal Skills Programme; 2018. Available from: https://casp-uk.net/casp-tools-checklists/. |
| 12 | Appraisal process | Indicate whether the appraisal was conducted independently by more than one reviewer and if consensus was required. | Appraisal conducted independently by two reviewers. Consensus was sought.  See “Quality appraisal” (Method, pp.20-21). |
| 13 | Appraisal results | Present results of the quality assessment and indicate which articles, if any, were weighted/excluded based on the assessment and give the rationale. | No articles were excluded on the basis of quality assessment alone. See “Quality appraisal (Results, p.20-21; and Table. Quality appraisal). |
| 14 | Data extraction | Indicate which sections of the primary studies were analysed and how were the data extracted from the primary studies? *(e.g. all text under the headings “results /conclusions” were extracted electronically and entered into a computer software).* | The whole manuscript was read and verbatim findings related to Transgender youth and healthcare were uploaded to NVivo. See “Data extraction and synthesis (Method, p. 9). |
| 15 | Software | State the computer software used, if any. | QSR International’s NVivo 11 Software. See “Data extraction and synthesis” (Method, p.9). |
| 16 | Number of reviewers | Identify who was involved in coding and analysis. | See “Data extraction and synthesis” (Method, p.8). |
| 17 | Coding | Describe the process for coding of data *(e.g. line by line coding to search for concepts).* | See “Data extraction and synthesis” (Method, pp.9-10). |
| 18 | Study comparison | Describe how were comparisons made within and across studies *(e.g. subsequent studies were coded into pre-existing concepts, and new concepts were created when deemed necessary).* | See “Data extraction and synthesis” (Method, p.9-10). |
| 19 | Derivation of themes | Explain whether the process of deriving the themes or constructs was inductive or deductive. | Inductive, see “Data extraction and synthesis” (Method, p.9-10). |
| 20 | Quotations | Provide quotations from the primary studies to illustrate themes/constructs,and identify whether the quotations were participant quotations of the author’s interpretation. | Quotes from participants were drawn from the included studies and used to illustrate themes and used throughout the finding section (See Results, pp23-35). |
| 21 | Synthesis output | Present rich, compelling and useful results that go beyond a summary of the primary studies (e.g. *new interpretation, models of evidence, conceptual models, analytical framework, development of a new theory or construct).* | See Results (pp.23-35). Details of each study’s contribution to themes is detailed in table. Line of argument 34-45/ |
