# Supplementary material for: Experiences of transgender and non-binary youth accessing gender-affirming care: A systematic review and meta-ethnography
Source: PLoS One. 2021 Sep 10;16(9):e0257194. doi: 10.1371/journal.pone.0257194 (PMC8432766; doi:10.1371/journal.pone.0257194)
Supplement: S2 Table — (DOCX) [file pone.0257194.s005.docx]

## **S5A: Gender Identities (2005-2020)**

| **Gender Identity of Young Participants - All Studies 2005-2020** | | | | | | |
| --- | --- | --- | --- | --- | --- | --- |
| **Study** | **Author** | **TM/M** | **TF/F** | **GNC** | **Other** | **Comment** |
| Study 1: | Breland et al. (2019) | 20 | 14 | 2 | - | (32 completed questionnaire, 10 of same were interviewed) Author did not specify gender identity of interviewees. |
| Study 2: | Clark et al. (2020) | 8 | 8 | 5 | - |  |
| Study 3: | Carlile.(2019) | - | - | - | - | Two thirds of youth were assigned sex at birth female – no further details on gender identity specified by author |
| Study 4: | Corliss et al. (2007) | - | 18 | - | - |  |
| Study 5: | Eisenberg et al. (2019) | 3 | 1 | 7 | 1 |  |
| Study 6: | Gridley et al. (2016) | 7 | 3 | 5 | - |  |
| Study 7: | Riggs et al. (2019) | 5 | 4 | 1 | - |  |
| Study 8: | Sansfacon et al. (2019) | 14 | 20 | 1 |  |  |
| Study 9: | Sperber et al. (2005) | 8 | 5 | 1 |  |  |
| Study 10: | Turban et al. (2017) | - | - | - | - | No gender identities detailed - mentions that "group of transgender and gender non-conforming youth included". |
| **Total** | | **65** | **73** | **22** | **1** |  |

## **S5B: Gender Identities (2016-2020)**

| **Gender Identity of Young Participants - All Studies 2016-2020** | | | | | | |
| --- | --- | --- | --- | --- | --- | --- |
| **Study** | **Author** | **TM/M** | **TF/F** | **GNC** | **Other** | **Comment** |
| Study 1: | Breland et al. (2019) | 20 | 14 | 2 | - | (32 completed questionnaire, 10 of same were interviewed) Author did not specify gender identity of interviewees. |
| Study 2: | Clark et al. (2020) | 8 | 8 | 5 | - |  |
| Study 3: | Carlile.(2019) | - | - | - | - | Two thirds of youth were assigned sex at birth female – no further details on gender identity specified by author |
| Study 5: | Eisenberg et al. (2019) | 3 | 1 | 7 | 1 |  |
| Study 6: | Gridley et al. (2016) | 7 | 3 | 5 | - |  |
| Study 7: | Riggs et al. (2019) | 5 | 4 | 1 | - |  |
| Study 8: | Sansfacon et al. (2019) | 14 | 20 | 1 |  |  |
| **Total** | | **57** | **50** | **21** |  |  |
| **Total including Carlile (2019)** |  | **75** | **50** | **25** |  | |
| **N= approx. 22 more estimated** |  |  |  |  |  |  |
| **Approx. n=4 GNC** |  |  |  |  |  |  |
