# Supplementary material for: Experiences of transgender and non-binary youth accessing gender-affirming care: A systematic review and meta-ethnography
Source: PLoS One. 2021 Sep 10;16(9):e0257194. doi: 10.1371/journal.pone.0257194 (PMC8432766; doi:10.1371/journal.pone.0257194)
Supplement: Supplementary file 6 [file pone.0257194.s002.odt]

## S3: Search Strategy

MESH/THEASURUS (MJMESH.EXACT("Transgender Persons") OR MJMESH.EXACT("Health Services for Transgender Persons"))

# Population

 OR ti(Transgender OR gender varian* OR gender conform* OR gender nonconform* OR gender identit* OR gender fluid* OR gender flex* OR gender dysphori* OR gender express* OR gender atypical OR gender bend* OR gender creative* OR gender independen* OR intersex* OR two-spirit OR genderqueer OR non binary OR non-binary OR MtF OR FtM OR transexual* ) 

AND ab(adolescen* OR youth* OR teen* OR tween* OR pubert* OR young adult* OR young people OR young person OR emerg* adult* OR child* OR children OR paediatric OR pediatric OR teen OR school age OR school aged OR high school OR secondary school OR juvenile ) 

# Exposure

 AND ab(healthcare OR health* OR health service* OR healthcare delivery OR health service accessibility OR primary health* OR hospital* OR gender care OR gender clinic* OR gender service OR gender reaffirm* OR gender affirm* OR hormone therap* or gender surger* OR transgender healthcare OR trans* health* OR trans healthcare OR transitional care)

# Outcome 

AND ab(Patient* experience* OR patient* perception* OR patient* perspective* OR patient* satisfaction* OR patient* dissatisfaction OR barrier* or enabler* or facilitator*)
