# Supplementary material for: Experiences of transgender and non-binary youth accessing gender-affirming care: A systematic review and meta-ethnography
Source: PLoS One. 2021 Sep 10;16(9):e0257194. doi: 10.1371/journal.pone.0257194 (PMC8432766; doi:10.1371/journal.pone.0257194)
Supplement: Supplementary file 7 [file pone.0257194.s004.odt]

## S4: CASP Scoring

| CASP Scoring - Systematic Review 2020 |
| --- |
| Yes, no, unclear | CASP Criterion 1 | CASP Criterion 2 | CASP Criterion 3 | CASP Criterion 4 | CASP Criterion 5 | CASP Criterion 6 | CASP Criterion 7 | CASP Criterion 8 | CASP Criterion 9 | CASP Criterion 10 |  |
| Citation | Clear statement of aim | Qualitative methodology appropriate | Appropriate research design | Sampling | Data collection | Research reflexivity | Ethical consideration | Appropriate data analysis | Clear statement of findings | Research value |  |
| Breland et al. (2019) | YES | YES | UNCLEAR | UNCLEAR | UNCLEAR | NO | YES | YES | YES | YES |  |
| Clark et al. (2020) | YES | YES | YES | YES | YES | NO | YES | YES | YES | YES |  |
| Carlile (2019) | YES | YES | YES | UNCLEAR | YES | YES | YES | YES | YES | YES |  |
| Corliss et al. (2007) | YES | YES | UNCLEAR | YES | YES | NO | YES | YES | YES | YES |  |
| Eisenberg et al. (2019) | YES | YES | UNCLEAR | YES | UNCLEAR | NO | YES | UNCLEAR | YES | YES |  |
| Gridley et al. (2016) | YES | YES | YES | YES | YES | NO | YES | YES | YES | YES |  |
| Riggs et al. (2019) | YES | YES | UNCLEAR | YES | YES | NO | YES | YES | YES | YES |  |
| Sansfacon et al. (2019) | YES | YES | YES | YES | YES | YES | YES | YES | YES | YES |  |
| Sperber et al. (2005) | YES | YES | YES | YES | YES | YES | YES | YES | YES | YES |  |
| Turban et al. (2017) | YES | YES | UNCLEAR | YES | UNCLEAR | YES | UNCLEAR | UNCLEAR | YES | YES |  |

																=
